# Progress of Temperance

**Published:** 1843-01

**Authors:** 


### PROGRESS OF TEMPERANCE.

We hope all our readers take an interest, and "lend a hand," in the extirpation of alcoholic intemperance, as one of the most fruitful sources of the diseases they are called to encounter. Our observations during the past summer, have convinced us, that there is a great and wide spread reduction in the quantity of alcohol lately consumed in this country. The reform, after having exerted its power in our garrisons to the north, has extended even among the Indians, many of whom have become total abstinents. In regard to the soldiers, Dr. Tripler, of Detroit barracks, informed us, in the month of August, that, from the 1st of November, 1841, to the 29th of April, 1842, a period of six months, through which the average number was two hundred and seventy-seven, there were six deaths, two from delirium tremens, and a third from general intemperate habits, making half of the whole; but from the 1st of May, to the 18th of August, there had not been a death. Temperance societies are now established in most of our garrisons, and in nearly every village of the whole country. Surely a signal abatement of disease will follow such a reform. D.

